# Oral Bioavailability Enhancement and Anti-Fatigue Assessment of the Andrographolide Loaded Solid Dispersion

**DOI:** 10.3390/ijms21072506

**Published:** 2020-04-04

**Authors:** Ching-Chi Yen, Yu-Kai Liang, Chao-Pei Cheng, Mei-Chich Hsu, Yu-Tse Wu

**Affiliations:** 1 School of Pharmacy, Kaohsiung Medical University, Kaohsiung 807, Taiwan; date0315@hotmail.com (C.-C.Y.); apccac1004@gmail.com (Y.-K.L.); hk810547@gmail.com (C.-P.C.); 2 Department of Sports Medicine, Kaohsiung Medical University, Kaohsiung 807, Taiwan; meichich@kmu.edu.tw; 3 Department of Medical Research, Kaohsiung Medical University Hospital, Kaohsiung 807, Taiwan; 4 Substance and Behavior Addiction Research Center, Kaohsiung Medical University, Kaohsiung 807, Taiwan; 5 Drug Development and Value Creation Research Center, Kaohsiung Medical University, Kaohsiung 807, Taiwan

**Keywords:** andrographolide, solid dispersion, oral bioavailability, anti-fatigue

## Abstract

Andrographolide (AG), a major diterpene lactone isolated from *Andrographis paniculata* (Burm. f.) Nees (Acanthaceae), possesses a wide spectrum of biological activities. However, its poor water solubility and low bioavailability limit its clinical application. Therefore, this study aimed to develop a solid dispersion (SD) formulation to increase the aqueous solubility and dissolution rate of AG. Different drug-polymer ratios were used to prepare various SDs. The optimized formulation was characterized for differential scanning calorimetry, Fourier transform infrared spectroscopy, and powder X-ray diffraction. The analysis indicated that the optimized SD enhanced AG solubility and dissolution rates by changing AG crystallinity to an amorphous state. The dissolution behaviors of the optimum SD composed of an AG-polyvinylpyrrolidone K30-Kolliphor EL ratio of 1:7:1 (*w*/*w*/*w*) resulted in the highest accumulated dissolution (approximately 80%). Pharmacokinetic studies revealed that *C*_max_/dose and the AUC/dose increased by 3.7-fold and 3.0-fold, respectively, compared with AG suspension. Furthermore, pretreatment using the optimized AG-SD significantly increased the swimming time to exhaustion by 1.7-fold and decreased the plasma ammonia level by 71.5%, compared with the vehicle group. In conclusion, the optimized AG-SD formulation appeared to effectively improve its dissolution rate and oral bioavailability. Moreover, the optimized AG-SD provides a promising treatment against physical fatigue.

## 1. Introduction

*Andrographis paniculata* (Burm. f.) Nees (Acanthaceae) is a medicinal plant native to south Asia and India. *A. paniculata* (AP) has a very bitter taste and is traditionally applied to ease internal heat, pain, and inflammation according to Chinese medicinal theory [1]. Andrographolide (AG) is a major diterpene lactone isolated from AP. Several studies have discovered the pharmacological activities of AG, and these findings demonstrated that AG might have potential for the alleviation of several diseases caused by the oxidative stress, and the main mechanisms include the activation of nuclear factor erythroid-2-related factor 2 and inhibition of nuclear factor-κB activation [2,3,4]. Recent studies have demonstrated that antioxidant supplementation may serve as a practical strategy for the alleviation of physical fatigue through modulating oxidative stress and inflammation status [5,6,7]. For example, the well-known anti-inflammatory curcumin and curcuminoids, which are hydroxycinnamic acid derivatives with polyphenolic rings, have demonstrated the ability to boost exhaustive swimming time and bust exercise fatigue-associated markers, including lactate, ammonia, and blood urea nitrogen (BUN) in mice [8]. In addition, one study enrolled 25 patients with multiple sclerosis and claimed that a 12-month supplementation of AP extract significantly reduced Fatigue Severity Scale scores compared with the placebo group [9]. AP has demonstrated positive effect in terms of relieving fatigue; however, the supplement period requires 12 months. Chen et al. [10] revealed that the oral bioavailability of AG was around 1.19% after a single administration (50 mg/kg), while our previous work has indicated the oral bioavailability of AG is approximate 0.3% after a single oral administration (300 mg/kg) [11]. Another study has found that the bioavailability of AG is 0.91% after a single 20 mg/kg administration and only 0.21% at a higher dose of 200 mg/kg [12]. Lee et al. [13] noted that AG belongs to biopharmaceutical classification system (BCS) class II compound, which is sparingly soluble in water. Several studies also revealed that AG was a substrate of P-glycoprotein (P-gp) [14,15]. Therefore, the low solubility (0.07 mg/mL) and the efflux transport by P-gp may lead to the poor oral absorption of AG. Thus, proposing a practical delivery system for increasing the oral bioavailability of AG remains crucial for the successful clinical and health application of this active component.

Various formulations have been proposed to improve the solubility, dissolution, and bioavailability of AG. These formulation approaches for AG included solid dispersion (SD) [16,17,18,19,20], liposomes [21], niosomes [22], and solid lipid nanoparticles (SLN) [23] and focused on in vitro characterization for delivery systems and assessing their effects on diverse cell models. The inclusion complex composed of AG and hydroxypropyl-β-cyclodextrin has been proposed to elevate the bioavailability of AG by 1.6-fold [14]. In addition, a pH-sensitive nanoparticle has been designed to improve the oral bioavailability of AG by approximately 2.2 times [24]. SLN has been proposed for enhancing the oral bioavailability of AG by approximately 2.41 times [25]. A liquid self-microemulsifying drug delivery system containing AG extract, Capryol 90, Kolliphor RH 40, and Labrasol has been developed, and the results revealed a 15-fold enhancement of absorption compared with that of the AG extract suspension [26]. However, a high emulsifier concentration in the formulation might result in gastrointestinal irritation, and stability is also crucial to ensure its feasibility.

The SD technique features as one of the more fascinating among solubilization methods because the combination of the active components and polymers with various functions can lead to significant enhancements in solubility, disintegration, and dissolution of active components [27]. Bothiraja et al. [16] demonstrated the utility of SD to improve the pharmaceutical properties of AG using polyvinylpyrrolidone (PVP) K-30 as a carrier. Zhao et al. [17] prepared AG-SD by spray-drying and vacuum-drying methods with different grafted polyethylene glycol (PEG) as carrier material. Zhang et al. [18] also confirmed that the feasibility of silica as a carrier of SD to enhance the dissolution of AG. However, pharmacokinetic data for the above systems are lacking; thus, their effects on the absorption of AG cannot be confirmed. Nanocrystal-based SD prepared by Ma et al. [19] and AG-solid self-nanodispersion delivery system developed by Xu et al. [20] have exhibited a fast dissolution rate and significantly improved the bioavailability of AG. Nevertheless, several steps containing homogenization and spray-drying technology render the optimization of their preparation process complicated and time-consuming. Thus, identifying a suitable drug delivery system with a relatively easy way for increasing the oral bioavailability of AG remains a task of great interest. Fitriani et al. [28] reported that SD can enhance the solubility of usnic acid and its antioxidant activity due to the improvement of usnic acid’s solubility. Nadal et al. [29] also proved that SD could retain the antioxidant activity of ferulic acid and further improved its in vivo anti-platelet effect. In addition, our pervious study has revealed that the anti-fatigue efficiency of resveratrol can be enhanced through dosage form modification [30]. Furthermore, there has been relatively little research conducted on anti-fatigue pharmacological effects after AG supplement. When composition parameters, including polymer types, molecular weight, and percentage within formulation, as well as the active component’s crystalline-amorphous states, are carefully manipulated, oral bioavailability improves considerably [31]. In the current study, we selected PVP and PEG with different average molecular weights to prepare an AG-loaded SD delivery system. The proposed optimized formulation was characterized in terms of its physicochemical properties, oral bioavailability, and potential anti-fatigue effects.

## 2. Results and Discussion

### 2.1. Formulation Screening by Dissolution

**Figure 1:**
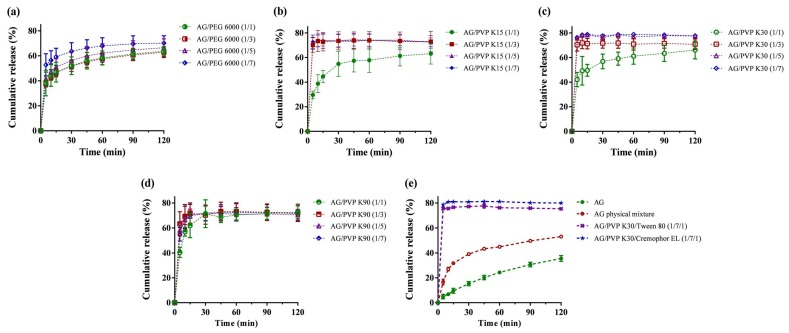
Dissolution profiles of andrographolide (AG) prepared using: (**a**) PEG 6000; (**b**) polyvinylpyrrolidone (PVP) K15; (**c**) PVP K30; (**d**) PVP K90; and (**e**) PVP K30 containing Tween 80 or Kolliphor EL as surfactant carrier. Data are presented as mean ± standard deviation (*n* = 3 for each group).

AG dissolution profiles, physical mixtures, and AG-loaded SDs were evaluated in 0.1 N HCl (pH 1.2) medium, as depicted in Figure 1. Dissolution performance parameters, including cumulative percentage release in 5 min (Q_5min_), cumulative percentage release in 120 min (Q_120min_), dissolution efficiency (D.E.), and the time required to reach 70% release (t_70%_), are summarized in Table 1. From the dissolution results (Figure 1e), AG demonstrated a relatively low accumulated dissolution of approximately 35%. Q_120min_ obviously increased to more than 75% when formulated as SD, and the physical mixture group (F20) displayed a Q_120min_ of approximately 53%. AG is recognized as a BCS class II compound [13], which means dissolution in the gastrointestinal tract is the rate-limiting step for oral absorption. Therefore, enhancing dissolution is crucial for efficiently improving the oral bioavailability of AG. Hydrophilic polymers, such as PEG and PVP, have been commonly used as carriers for SD because of their low cost, sufficient water solubility, and ability to transform active ingredients into amorphous states [32]. The dissolution profile revealed that the SD made of PVP generally exhibited a quicker release than SD made of PEG 6000 (Figure 1a). When the AG/polymer ratio is greater than 1:3 in the formulations of PVP K15 (Figure 1b) and K30 (Figure 1c), the average values of Q_5min_ could surpass 70%. However, the dissolution pattern of AG from PVP K90 SD (Figure 1d) was slower (Q_5min_ < 63.3%). This phenomenon may have resulted from the elevated local viscosity caused by PVP K90, which restricts the diffusion near the boundary layer of SD [33]. We selected F13 (AG/PVP K30 1:7, w/w) for further investigation because it displayed the highest Q_5min_, Q_120min_, and D.E. The effects of surfactant addition on dissolution were also examined, and the addition of Kolliphor EL brought the accumulated dissolution to approximately 80%. Finally, we chose F19 (AG/PVP K30/Kolliphor EL = 1:7:1, *w*/*w*/*w*) as the optimized formulation. Incorporation of a surfactant in a SD to improve the dissolution rate is also described in other studies [34,35]. These results may be explained as the inclusion of surfactants in the formulation containing a polymeric carrier might help to prevent precipitation and/or protect a fine crystalline precipitate from agglomeration into much larger hydrophobic particles [36].

### 2.2. Differential Scanning Calorimetric Analysis

**Figure 2:**
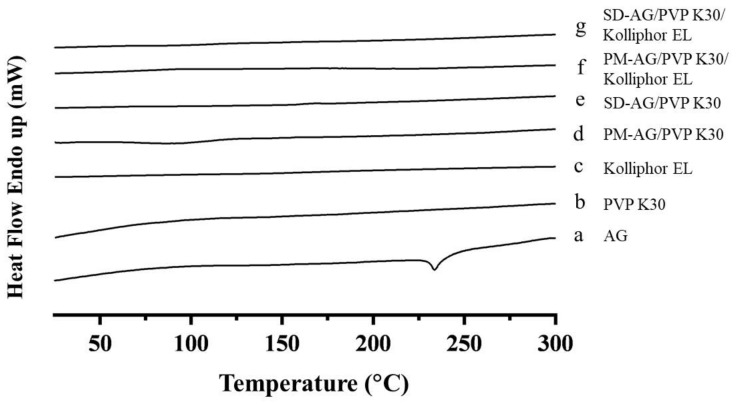
Differential scanning calorimetry spectrum of: (**a**) andrographolide (AG); (**b**) PVP K30; (**c**) Kolliphor EL; (**d**) physical mixture (PM)-AG/PVP K30 (1:7, *w*/*w*); (**e**) solid dispersion (SD)-AG/PVP K30 (1:7, w/w); (**f**) PM-AG/PVP K30/Kolliphor EL (1:7:1, *w*/*w*/*w*); and (g) SD-AG/PVP K30/Kolliphor EL (1:7:1, *w*/*w*/*w*).

The differential scanning calorimetry (DSC) analysis was used to assess the amorphous state of AG. Pure AG displayed an endothermic peak at 233 °C (Figure 2a), which corresponded to the melting point of AG [37]. Figure 2b,c showed the thermograms of PVP K30 and Kolliphor EL. For both SD formulations (Figure 2e,g), the endothermic peak of AG at approximately 233 °C disappeared and no other endothermic peaks were observed, implying AG mainly existed in amorphous state in the solid. AG prepared by physical mixture combinations (Figure 2d,f) also displayed a disappearance of sharp AG endothermic peak; this phenomenon may be caused by the dilution of AG sample via the preparation process. It could not be proved that AG was under its amorphous form. This was further confirmed by powder X-ray diffraction (XRPD) study.

### 2.3. Powder X-Ray Diffraction Analysis

**Figure 3:**
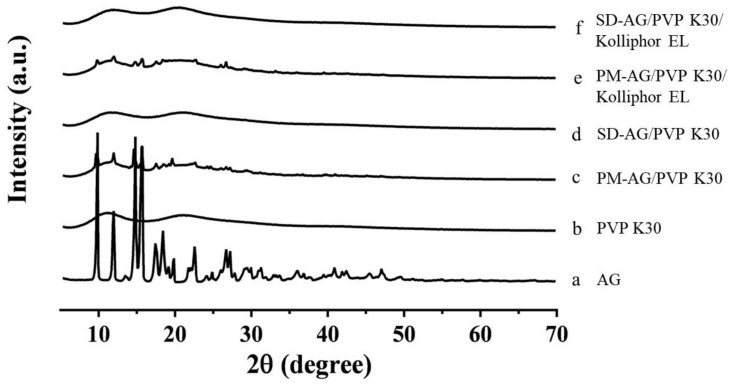
Powder x-ray diffraction patterns of: (**a**) andrographolide (AG); (**b**) PVP K30; (**c**) physical mixture (PM)-AG/PVP K30 (1:7, *w*/*w*); (**d**) solid dispersion (SD)-AG/PVP K30 (1:7, *w*/*w*); (**e**) PM-AG/PVP K30/Kolliphor EL (1:7:1, *w*/*w*/*w*); and (**f**) SD-AG/PVP K30/Kolliphor EL (1:7:1, *w*/*w*/*w*).

PXRD patterns of pure AG, PVP K30, physical mixtures, and AG-loaded SDs are illustrated in Figure 3. As depicted in Figure 3a, AG’s diffraction pattern was highly crystalline in its natural state, as indicated by the numerous peaks. The results resemble those of Ma et al. [19]. The powdered PVP K30 was amorphous where no prominent peaks were detected (Figure 3b). The physical mixtures displayed diffraction patterns similar to those of pure AG, indicating that the crystallinity of AG was not affected by the process utilized to obtain it (Figure 3c,e). However, the powder X-ray diffraction patterns of the SDs suggested that AG was no longer present in its crystalline form in the SDs but existed in an amorphous state (Figure 3d,f). These results confirmed that AG was present in amorphous form within the SDs.

### 2.4. Fourier Transform Infrared Spectroscopy

**Figure 4:**
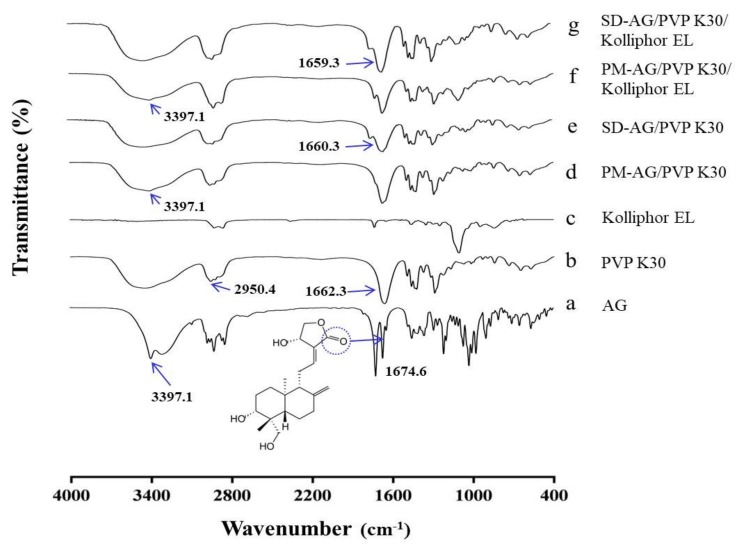
Fourier-transform infrared spectroscopy spectrums of: (**a**) andrographolide (AG); (**b**) PVP K30; (**c**) Kolliphor EL; (**d**) physical mixture (PM)-AG/PVP K30 (1:7, *w*/*w*); (**e**) solid dispersion (SD)-AG/PVP K30 (1:7, *w*/*w*); (**f**) PM-AG/PVP K30/Kolliphor EL (1:7:1, *w*/*w*/*w*); and (**g**) SD-AG/PVP K30/Kolliphor EL (1:7:1, *w*/*w*/*w*).

The Fourier transform infrared spectroscopy (FT-IR) spectroscopy was applied to evaluate the possible chemical interactions between AG and PVP. AG demonstrated a typical C=O band at 1674.6 cm^−1^ and an OH stretch at 3397.1 cm^−1^ (Figure 4a), which corresponded to the results of a previous report [20]. The characteristic bands of PVP appeared approximately at 1662.3 and 2950.4 cm^−1^ (Figure 4b), which corresponded to the pyrrole moiety and the C–H stretch, respectively [38]. Spectra characteristic band from 1550 to 1650 cm^−1^, which is typically observed for carboxylate anions, was not observed in the purified Kolliphor EL (Figure 4c) [39]. The FTIR spectra of physical mixtures (Figure 4d,f) seemed to be only a summation of AG and PVP K30 spectra, implying a lack of chemical interaction. For the formulations of SD, the OH stretch at 3397.1 cm^−1^ disappeared, and the C=O band at 1674.6 cm^−1^ shifted to 1660.3 (Figure 4e) and 1659.3 cm^−1^ (Figure 4g). These results could be explained by intermolecular interactions (e.g., hydrogen bonding), suggesting that the crystalline form of AG was transformed into an amorphous state [40].

### 2.5. Ex vivo Permeability

**Figure 5:**
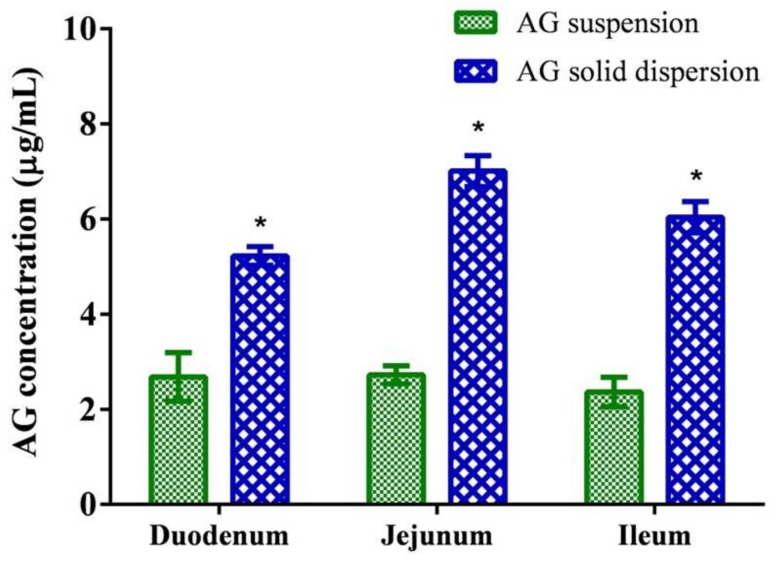
Permeability test of andrographolide (AG) suspension and optimized AG-loaded solid dispersion. Data are presented as mean ± standard deviation (*n* = 3 for each group). * Indicates significant difference compared to the AG suspension group (*p* < 0.05).

The concentrations of AG absorbed from the suspension and the optimized SD through intestinal segments are depicted in Figure 5. The results indicate that the AG concentrations in the duodenum, jejunum, and ileum were 2.55 ± 0.49, 2.56 ± 0.36, and 2.18 ± 0.45 μg/mL, respectively, and the optimized AG-loaded SD composed of AG/PVPK30/Kolliphor EL (1:7:1, *w*/*w*/*w*) significantly enhanced the penetration of AG at intestinal segments. The jejunum proved to be the best region for AG absorption in SD, which attained a concentration approximately 2.7 times higher than that of the unformulated AG suspension. The results suggest that the optimized formulation improved dissolution performance translated to higher intestinal permeability of AG.

### 2.6. Oral Bioavailability Assessment

**Figure 6:**
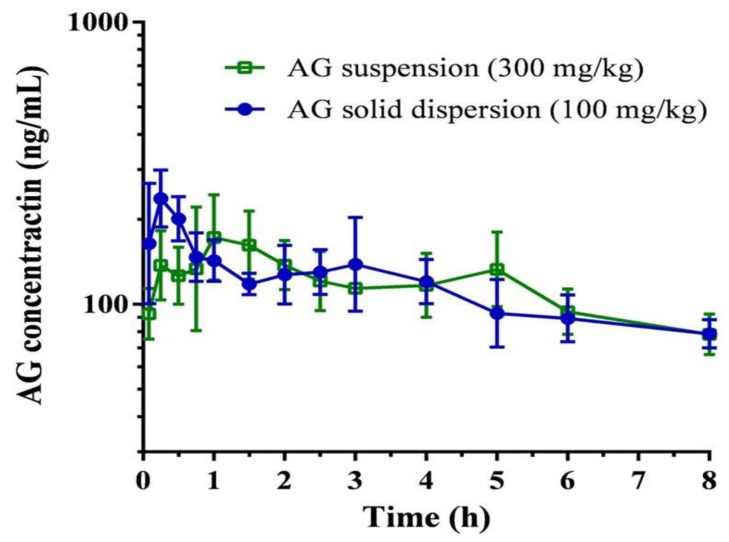
Plasma concentration-time curve of andrographolide (AG) after oral administration of AG suspension and optimized AG-loaded solid dispersion in rats. Data are expressed as mean ± standard deviation (*n* = 5 for each group).

The plasma concentration–time curve and pharmacokinetic parameters of AG after oral administration of the AG suspension (300 mg/kg) and the optimized AG-loaded SD (100 mg/kg,) in rats are depicted in Figure 6 and Table 2, respectively. The maximum plasma concentration (*C*_max_)/dose and the area under the plasma concentration–time curve (AUC)/dose attained after administration of the AG-loaded SD were 2.5 ± 0.6 rat ng/mL and 9.3 ± 1.8 h rat ng/mL, which were 3.7-fold and 3.0-fold higher than those of the oral AG suspension, respectively. The relative bioavailability was determined to be 297.7%. These data provide strong evidence for improvement in AG absorption under optimized AG-loaded SD. AG possesses low solubility and encounters extensive metabolism together with efflux transportation by P-gp in the intestinal tract, which contributes to limited oral absorption. A higher dose of 300 mg/kg was therefore selected to appropriately reflect the pharmacokinetic profile of AG suspension. According to a previous study [41], Kolliphor EL, which might be P-gp specific, could inhibit the function of P-gp, thereby increasing the intestinal absorption of various drugs that are secreted by a P-gp-mediated efflux system in the intestine. Therefore, the use of Kolliphor EL in the SD might prevent the efflux of AG by P-gp to increase AG oral absorption.

### 2.7. Anti-Fatigue Effects

#### 2.7.1. Physical Performance Test

**Figure 7:**
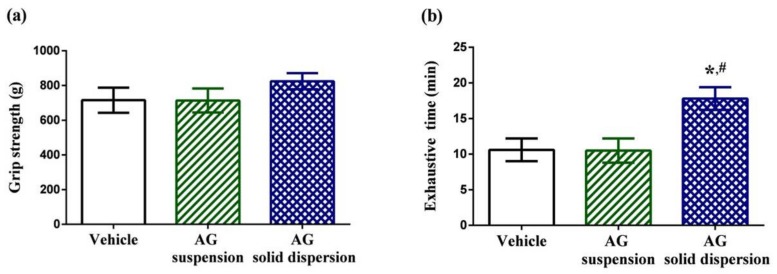
Effect of andrographolide (AG) on: (**a**) forelimb grip test; and (**b**) exhaustive swimming test. Data are expressed as mean ± standard deviation (*n* = 5 for each group). * Indicates significant difference compared to vehicle group (*p* < 0.05). ^#^ Indicates significant difference compared to AG suspension group (*p* < 0.05).

The physical performance tests involved a forelimb grip test and exhaustive swimming test. Grip strength tests can be used to assess motor-associated coordination and adaption in neurological studies and reflect the overall health of the musculoskeletal system. Grip strength in the vehicle, AG suspension, and AG-loaded SD group were 715.2 ± 72.7, 713.8 ± 69.2, and 824.4 ± 45.8 g, respectively (Figure 7a). We discovered no significant differences in grip strength among the three treatment groups. The exercise endurance test is a major method in evaluating anti-fatigue treatment. The exhaustive swimming time in the vehicle, AG suspension, and AG-loaded SD groups were 10.6 ± 1.6, 10.5 ± 1.7, and 17.8 ± 1.6 min, respectively (Figure 7b). Our data indicate that pretreatment with AG suspension did not increase the exhaustive swimming time, which might be explained by the low AG absorption. By contrast, the exhaustive swimming time was prolonged by 1.7-fold with AG-loaded SD compared with that in the vehicle group.

Fatigue can be defined as a state of weariness that is either physical or mental, which is caused by exertion and can lead to deterioration in physical performance [42]. According to previous studies, oxidative stress and inflammatory mediators could be possible weighty mechanisms for physical fatigue because the accumulation of reactive oxygen species during exercise processes raises oxidative stress and leads to oxidative damage on mitochondria and muscle contractile protein [43]. As an antioxidant, AP has proven its ability to relieve fatigue; however, the pretreatment protocols are time-consuming, possibly due to its low bioavailability. As a result, the optimized AG-loaded SD overcomes the shortcomings of time-consuming AG pretreatment due to the enhancement of AG oral bioavailability. Moreover, the anti-fatigue potential of AG-loaded SD has also been demonstrated.

#### 2.7.2. Lactate Production and Clearance Ratio

**Figure 8:**
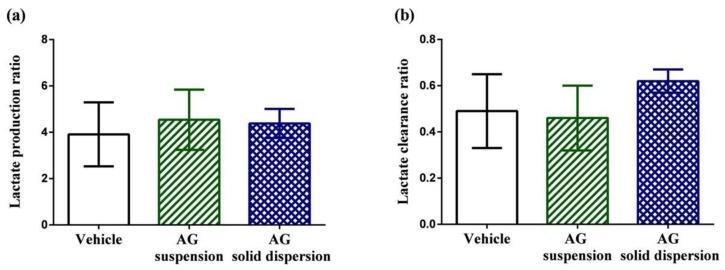
Effect of andrographolide (AG) on: (**a**) lactate production ratio; and (**b**) lactate clearance ratio. Data are expressed as mean ± standard deviation (*n* = 5 for each group). Lactate production ratio = (Lactate_post-swimming_ − Lactate_pre-swimming_)/Lactate_pre-swimming_. Lactate clearance ratio = (Lactate_post-swimming_ − Lactate_30 min later after post-swimming_)/Lactate_post-swimming_.

Figure 8a,b displays lactate production and clearance ratios during high-intensity swimming. Anaerobic glycolysis is the transformation of glucose to lactate when limited amounts of oxygen are available. During high-intensity exercise, the intramuscular accumulation of lactic acid has long been considered one of the most crucial factors in fatigue [44]. The results show that the lactate production and clearance ratios among the three groups did not show significant difference.

#### 2.7.3. Fatigue-Related Indicator Determination

**Figure 9:**
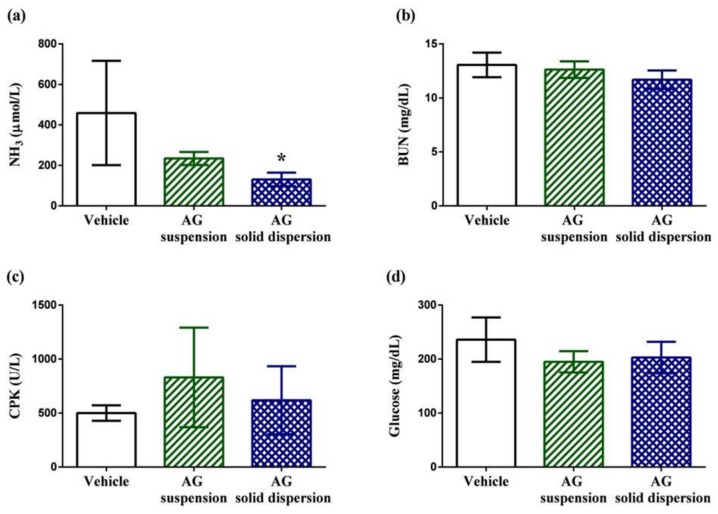
Effect of andrographolide (AG) on: serum (**a**) ammonia; (**b**) blood urea nitrogen (BUN); (**c**) creatine phosphokinase (CPK); and (**d**) glucose levels after swimming challenge. Data are expressed as mean ± standard deviation (*n* = 5 for each group). * Indicates significant difference compared to vehicle group (*p* < 0.05).

Biochemical indicators containing plasma ammonia (NH_3_), blood urea nitrogen (BUN), creatine phosphokinase (CPK), and glucose are normally used to assess the status of muscle fatigue after exercise [45]. Plasma ammonia levels in the vehicle, AG suspension, and AG-loaded SD groups were 459 ± 258, 234 ± 32, and 131 ± 33 μmol/L, respectively (Figure 9a). Ammonia, a metabolite of protein and amino acid, is linked to neuromuscular fatigue during resistance training and affects continual coordinated activity in critical regions of the central nervous system [46,47]. The plasma ammonia levels in rats were significantly lower (by 71.5%) after pretreatment with the AG-loaded SD compared with those in the vehicle group. Urea nitrogen is the metabolic outcome of protein and amino acids. When carbohydrates and fats cannot provide sufficient energy to the body, BUN levels increase because of strong catabolic metabolism form protein and amino acids. Previous studies have revealed that BUN may have a positive correlation with exercise tolerance [48,49]. Therefore, BUN is another crucial biochemical parameter related to fatigue. The results reveal that the BUN level decreased by 10.7% after AG-loaded SD treatment; however, the values did not differ in both groups (Figure 9b). CPK is a clinical biomarker for muscle damage [50], and no significant differences in the activity of CPK among the three treated groups were observed, suggesting the inhibition of muscle damage after pretreatment with AG was not obvious (Figure 9c). Efficient utilization of glucose is an important performance maintenance index during exercise [51]. The results reveal that serum glucose level was lowered by 17.4% and 13.9% with AG suspension and AG-loaded SD treatments compared with the vehicle treatment group after exercise (Figure 9d). Although the values of serum glucose did not statistically differ among the three groups, AG appears to have the potential to make tissues more efficiently use glucose.

Supplementation with antioxidant nutrients could prevent exercise-induced oxidative damage and lactate accumulation [52,53]. As an antioxidant and owning anti-inflammatory activity, we hypothesized AG may serve as a practical strategy for the alleviation of physical fatigue via modulating oxidative stress and inflammation status. Our study indicated that AG-loaded SD supplements could prolong the exhaustive swimming time. To identify the evidence most relevant to the mechanism, exercise-induced fatigue-related parameters, such as lactate, ammonia, BUN, CPK, and glucose levels, were investigated. The results show that the improvement of exercise performance in rat might be explicated by reducing blood ammonia. According to a previous study, oxidative stress is an evolving concept in the pathogenesis of ammonia neurotoxicity [54]. As an antioxidant agent, AG could reduce blood ammonia, especially in SD forms that could enhance the bioavailability of AG.

#### 2.7.4. Tissue Glycogen Determination

**Figure 10:**
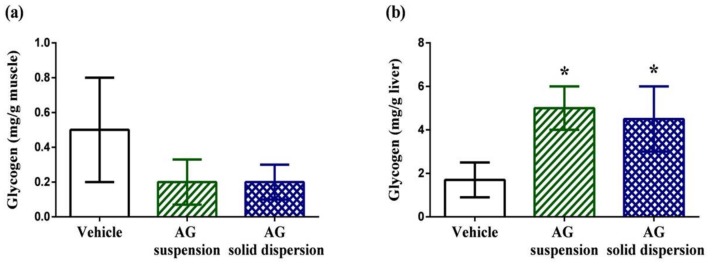
Effect of andrographolide (AG) on: glycogen content in: (**a**) muscle; and (**b**) liver. Data are expressed as mean ± standard deviation (*n* = 5 for each group). * Indicates significant difference compared to vehicle group (*p* < 0.05).

Muscular glycogen levels in the vehicle, AG suspension, and AG-loaded SD groups were 0.5 ± 0.3, 0.2 ± 0.13, and 0.2 ± 0.1 mg/g, respectively (Figure 10a). We did not find any significant difference in the muscular glycogen contents among groups. By contrast, hepatic glycogen levels in the vehicle, AG suspension, and AG-loaded SD groups were 1.7 ± 0.8, 5.0 ± 1.0, and 4.5 ± 1.5 mg/g, respectively (Figure 10b). Energy expenditure and deficiency can lead to physical fatigue during exercise. Therefore, energy storage and supply are another crucial factor related to exercise performance [55]. In this study, we discovered that hepatic glycogen level was significantly increased by 2.9-fold and 2.6-fold with AG suspension and AG-loaded SD treatments. The same phenomenon was also observed by Subramanian et al. [56]. They revealed that treatment with AG and its extract for 21 days increased glucokinase levels, which is the first step for both glycogen and glycolysis synthesis in the liver. The synthesis of glucokinase is highly sensitive to oxidative stress. As an antioxidant agent, AG increases liver glycogen levels, which explains the observed increase in glucokinase.

## 3. Materials and Methods

### 3.1. Chemicals and Reagents

Andrographolide (purity ≥ 99.0%), fisetin (purity ≥ 96.0%), Tween 80, PVP K15 (Mw 15,000 g/mol), PVP K30 (Mw 40,000 g/mol), and PVP K90 (Mw 360,000 g/mol) were obtained from Tokyo Chemical Industry (Tokyo, Japan). PEG 6000 was purchased from Koch-Light Laboratories Limited (Suffolk, UK). Kolliphor EL and ethanol (≥ 99.8%) were purchased from Sigma-Aldrich (St. Louis, MO, USA). Acetonitrile of chromatographic grade was purchased from Macron Fine Chemicals (Center Valley, PA, USA). Other chemicals used in the study were all of analytical grade. Pure water was obtained using the Milli-Q system (Millipore, Bedford, MA, USA).

### 3.2. HPLC Analysis of Andrographolide

The high-performance liquid chromatography (HPLC) system comprised an L-2130 pump, L-2200 auto autosampler, and L-2420 UV-VIS detector (Hitachi, Tokyo, Japan). AG was separated on a reversed-phase Luna C18 column (250 mm × 4.6 mm i.d., 5 μm, Phenomenex, Torrance, CA, USA). The mobile phase consisted of acetonitrile and ammonium phosphate solution (10 mM, pH 2.5 with phosphoric acid) at a ratio of 600:1500 (*v*/*v*) and the flow rate was set at 1.5 mL/min. The mobile phase was filtered (0.22 μm, Bedford, MA, USA) and subjected to sonication (Branson 3210, Brookfield, CT, USA) prior to use. A 20-μL volume was injected into the column, and AG was monitored at 225 nm. A stock solution of AG (1.0 mg/mL) was prepared in acetonitrile, and working standard samples were prepared using a serial dilution of the stock solution. The coefficient of determination (≥ 0.9995) was used to confirm the linear range covering 1.25–100 μg/mL.

### 3.3. Preparation of Andrographolide-Loaded Solid Dispersion and Physical Mixture

AG and polymers (PVP or PEG) combined at different ratios (e.g., 1:1, 1:3, 1:5, and 1:7, w/w) were processed using a solvent evaporation method [40]. Briefly, AG (50 mg) and polymer were dissolved in 10 mL ethanol, and the mixture was sonicated for 15 min. The mixture solution was dried using rotary evaporation (N-1000SW, EYELA, Tokyo, Japan) at 50 °C under reduced pressure. The residual ethanol was removed by placing the solution in an oven at 50 °C for 24 h. A physical mixture was prepared by mixing a corresponding amount of AG with PVP K30 and Kolliphor EL (1:7:1, *w*/*w*/*w*) using a porcelain mortar. Each formulation was sieved through a 40-mesh sieve to minimize particle size variation among samples. All formulations were kept in a glass desiccator with silica desiccant.

### 3.4. Formulation Characterization

#### 3.4.1. In vitro Dissolution

The in vitro dissolution study was performed according to the procedure of a previous work [57]. The release of AG from the physical mixture and SDs were evaluated using the paddle method (SR8 PLUS dissolution test station, Hanson Research, Chatsworth, CA, USA). The 0.1 N hydrochloric acid solution (pH 1.2, 900 mL) was used as the dissolution medium, which was maintained at 37 ± 0.5 °C with a stir speed of 100 revolutions per minute (rpm). The sample (1 mL) was collected at 0, 5, 10, 15, 30, 45, 60, 90, 120, 150, and 180 min, and the dissolution fluid was maintained by supplying an equal volume of fresh 0.1 N HCl solution after each withdrawal. The samples were centrifuged at 25 °C (10000 rpm for 10 min), and the supernatant was transferred to the autosampler vials for HPLC analysis. Parameters, such as Q_5min_, Q_120min_, D.E., and t_70%_, were used for evaluating the dissolution performance of the various formulations.

#### 3.4.2. Differential Scanning Calorimetric Analysis

DSC assessment was conducted by weighing a 10-mg sample in an aluminum pan heated from 25 °C to 300 °C (rate: 10 °C/min) using a differential scanning calorimeter (Model 404 F3, Netzsch, Bavaria, Germany).

#### 3.4.3. Powder X-Ray Diffraction Analysis

PXRD pattern of pure AG, polymer as well as the physical mixture and SD were recorded using an X-ray diffractometer (Siemens D5000, Munich, Germany). The samples were ground into a fine powder before analysis and determined over a 2θ range of 5°–70° with a scan step size of 0.1° at a scan speed of 0.76 s/step.

#### 3.4.4. Fourier Transform Infrared Spectroscopy

The samples were dispersed in potassium bromide using a mortar and pestle and were pressed to thin and translucent tablets. FT-IR was performed in a PerkinElmer 2000 spectrophotometer (Waltham, MA, USA), and the spectra were recorded from 4000 to 400 cm^−1^.

### 3.5. Animals

The protocol for animal experiments, based on the guidelines of the Animal Protection Act and A Guidebook for the Care and Use of Laboratory Animals published by the government authority and approved by the Institutional Animal Care and Use Committee of Kaohsiung Medical University Hospital (No. IACUC-105006, 22, Mar. 2017), were followed. Male Sprague-Dawley rats (weight: 200 ± 20 g) were obtained from the BioLASCO company (Taipei, Taiwan). Animals were specifically pathogen-free and were housed with a 12-h light-dark cycle and fed for 24 h with food and water.

### 3.6. Ex vivo Permeability

The permeability assessment for AG was conducted using the everted gut sac method [58]. Briefly, reverted intestine segments (duodenum, jejunum, and ileum) filled with Tyrode’s solution were transferred into flasks containing Tyrode’s solution. AG and the optimized AG-loaded SD were separately added to the flasks to achieve a concentration of 50 μg/mL of AG. Tyrode’s solution with each segment sample was collected after shaking in a water bath for 1 h, and AG content within was analyzed using the HPLC method.

### 3.7. Oral Bioavailability Evaluation

Two groups of male Sprague-Dawley rats (*n* = 5 for each) were involved in this study, and repeated blood sampling was achieved through a jugular vein catheterization model [59]. Both AG powder and the optimized AG-loaded SD were suspended in pure water. One group received AG (dose: 300 mg/kg), and the other group received AG-loaded SD (dose: 100 mg/kg). Blood samples (300 μL) were taken using a catheter at 0.083, 0.25, 0.5, 0.75, 1, 1.5, 2, 2.5, 3, 4, 5, 6, and 8 h after AG administration. For the HPLC analysis of rat plasma samples, AG was extracted and determined according to the procedure of our previous paper [11]. Oral bioavailability was calculated by comparing the AUC between AG suspension and AG-loaded SD after dosage normalization.

### 3.8. Anti-Fatigue Evaluation

The anti-fatigue evaluation for AG was conducted using swimming exercises according to our previous report [30] with slight modification. Three groups of male Sprague-Dawley rats (*n* = 5 for each) were used in this experiment and were given three different pretreatments for 3 days. The first group was orally given water and served as a vehicle group; the second group was orally given AG suspension (100 mg/kg) and served as the control group; and the third group was given AG-loaded SD (100 mg/kg). After a 3-day pretreatment with AG-loaded SD, the rats were individually placed in water containers (40 cm long, 40 cm wide, and 50 cm high) with water at a depth of 30 cm kept at 27 ± 1 °C to determine lactate production and clearance ratios, forelimb grip strength, blood biochemical variables, swimming exercise performance, and tissue glycogen levels.

For the exhaustion exercise test, weights equivalent to 15% of body weight were attached to the rats. Swimming time from beginning to exhaustion was recorded as the endurance of each rat. Time for exhaustion was defined as time taken for rats to experience a loss of movement coordination and inability to return to the surface within 7 s. For lactate production and clearance, rats were loaded with constant weights corresponding to 5% of individual body weight and were allowed to swim for 10 min. Lactate Pro™2 (LT-1730, Kyoto, Japan) was used to analyze pre-swimming and post-swimming blood lactate levels half an hour before and after swimming. The blood lactate production and clearance ratios were calculated using the following equations: Lactate production ratio = (Lactate_post-swimming_ − Lactate_pre-swimming_)/Lactate_pre-swimming_; Lactate clearance ratio = (Lactate_post- swimming_ − Lactate_30 min later after post-swimming_)/Lactate_post-swimming_. For measurement of forelimb grip strength in rats, a low-force testing system (BioSeb, Chaville, France) was utilized in this test. The effects of AG on NH_3_, BUN, CPK, and glucose were evaluated after a 15-min swimming exercise with 5% of individual body weight-loading. All blood biochemical levels were measured using an autoanalyzer (Beckman Coulter DXC800, Brea, CA, USA). For tissue glycogen determination, skeletal muscles and liver were used to investigate changes in their glycogen content after AG-loaded SD administration. The method used to measure and analyze glycogen content was the same as described in our previous studies [30].

### 3.9. Data Analysis

The experimental results are presented as mean ± standard deviation values. The pharmacokinetic parameters were derived from a noncompartmental model analysis [60,61]. SPSS v14.0 (SPSS Inc., Chicago, IL, USA) was used to conduct analysis of variance, and multiple comparisons of variance were evaluated using the Scheffe post hoc test. A *p* value of < 0.05 was considered significantly difference.

## 4. Conclusions

In this study, AG-loaded SD was successfully developed to improve the dissolution profile of AG and increase its oral absorption compared with free AG powder. The results suggest that the dissolution rate of AG from SD depended on the concentration of the carrier. Moreover, PVP generally exhibited quicker release than SD made of PEG 6000. PVP K30 inhibited the crystallization of the drug, resulting in the amorphous form of the drug in SD. PXRD and DSC results confirmed the amorphous state of the drug in SD. The pharmacokinetic analysis conducted on the rats indicated that the optimized AG-loaded SD (AG/PVP K30/Kolliphor EL = 1:7:1, *w*/*w*/*w*) increased the relative bioavailability of AG by approximately three-fold. Furthermore, we confirmed that the optimized SD could increase the swimming time to exhaustion by 1.7-fold, and positively modulate fatigue-related parameters, including ammonia values and liver glycogen levels. Thus, AG-loaded SD not only provides a promising strategy for improving AG oral bioavailability but also protection against physical fatigue.

## Figures and Tables

**Table 1:** Ingredients, weight ratios, and dissolution performance of andrographolide (AG) and AG-loaded solid dispersion formulations.

Formulation	Ingredients	Weight Ratio (*w*/*w*)	Dissolution			
			Q_5min_	Q_120min_	D.E.	t_70%_ (min)
1	AG	NA	4.9 ± 1.8	35.6 ± 2.3	22.4 ± 1.7	NA
2	AG, PEG 6000	1/1	38.2 ± 10.2 ^*^	63.7 ± 3.6 ^*^	55.1 ± 5.8 ^*^	NA
3	AG, PEG 6000	1/3	37.9 ± 2.7 ^*^	62.9 ± 4.3 ^*^	54.5 ± 4.2 ^*^	NA
4	AG, PEG 6000	1/5	41.5 ± 5.6 ^*^	66.6 ± 7.0 ^*^	58.7 ± 6.9 ^*^	NA
5	AG, PEG 6000	1/7	52.8 ± 8.9 ^*^	70.3 ± 5.9 ^*^	64.7 ± 6.5 ^*^	120
6	AG, PVP K15	1/1	29.6 ± 3.1 ^*^	63.2 ± 8.6 ^*^	54.7 ± 7.8 ^*^	NA
7	AG, PVP K15	1/3	70.2 ± 5.9 ^*^	73.0 ± 8.3 ^*^	71.9 ± 8.2 ^*^	5
8	AG, PVP K15	1/5	71.6 ± 4.9 ^*^	72.4 ± 3.6 ^*^	72.3 ± 4.6 ^*^	5
9	AG, PVP K15	1/7	72.9 ± 5.5 ^*^	72.8 ± 5.1 ^*^	71.8 ± 5.2^*^	5
10	AG, PVP K30	1/1	42.1 ± 5.8 ^*^	66.2 ± 7.3 ^*^	58.0 ± 6.2 ^*^	NA
11	AG, PVP K30	1/3	70.4 ± 5.1 ^*^	70.8 ± 4.3 ^*^	69.8 ± 4.2 ^*^	5
12	AG, PVP K30	1/5	75.9 ± 0.2 ^*^	77.5 ± 0.1 ^*^	75.9 ± 0.7 ^*^	5
13	AG, PVP K30	1/7	76.3 ± 0.9 ^*^	77.8 ± 1.4 ^*^	77.8 ± 1.7 ^*^	5
14	AG, PVP K90	1/1	40.5 ± 4.1^*^	72.5 ± 5.1 ^*^	66.9 ± 5.3 ^*^	30
15	AG, PVP K90	1/3	63.3 ± 9.7 ^*^	72.2 ± 6.9 ^*^	70.3 ± 7.0*	15
16	AG, PVP K90	1/5	58.3 ± 4.3 ^*^	70.6 ± 4.4 ^*^	68.9 ± 5.1 ^*^	30
17	AG, PVP K90	1/7	55.0 ± 5.4 ^*^	71.5 ± 6.3 ^*^	69.7 ± 6.4 ^*^	15
18	AG, PVP K30, Tween 80	1/7/1	75.7 ± 1.2 ^*^	75.4 ± 0.6 ^*^	74.8 ± 0.1 ^*^	5
19	AG, PVP K30, Kolliphor EL	1/7/1	78.7 ± 0.1 ^*^	80.0 ± 0.7 ^*^	78.9 ± 0.5 ^*^	5
20	Physical mixture (AG, PVP K30, Kolliphor EL)	1/7/1	16.7 ± 2.3 ^*^	53.2 ± 0.2 ^*^	43.2 ± 0.5 ^*^	NA

PVP, polyvinylpyrrolidone; PEG, polyethylene glycol; Q_5min_, cumulative percentage release in 5 min; Q_120min_, cumulative percentage release in 120 min; D.E., dissolution efficiency; t_70%_, the time required to reach 70% release; NA, not applicable. The results are presented as mean ± standard deviation (*n* = 3 for each formulation); ^*^ *p* < 0.05, compared with AG group

**Table 2:** Pharmacokinetic parameters of andrographolide (AG) after oral administration of AG suspension, and AG-loaded solid dispersion in rats.

Parameters	Units	AG Suspension (300 mg/kg, p.o.)	AG solid Dispersion (100 mg/kg, p.o.)
*T*_max_	h	2.1 ± 1.8	0.4 ± 0.3
*t*_1/2_	h	7.1 ± 3.7	7.0 ± 3.1
*C*_max_	ng/mL	206.6 ± 57.6	254.0 ± 59.7
AUC_0-t_	h ng/mL	935.3 ± 130.3	928.2 ± 181.1
*C*_max_/dose	(ng/mL)/(mg/kg)	0.7 ± 0.2	2.5 ± 0.6 ^*^
AUC_0-t_/dose	(h ng/mL)/(mg/kg)	3.1 ± 0.4	9.3 ± 1.8 ^*^
Relative bioavailability	%	-	297.7

*T*_max_, time of occurrence for maximum AG concentration; *C*_max_, maximum concentration of AG; *t*_1/2_, AG half-life; AUC_0-t_, AG area under the plasma concentration–time curve from zero (0) h to time (t). ^∗^ Significantly different compared to AG suspension group (*p* < 0.05). Data are expressed as mean ± standard deviation (*n* = 5 for each group).
